# Genetic risk prediction in Hispanics/Latinos: milestones, challenges, and social-ethical considerations

**DOI:** 10.1007/s12687-023-00686-4

**Published:** 2023-11-14

**Authors:** Betzaida L. Maldonado, Daniel G. Piqué, Robert C. Kaplan, Katrina G. Claw, Christopher R. Gignoux

**Affiliations:** 1 Human Medical Genetics & Genomics Graduate Program, University of Colorado-Anschutz Medical Campus, Aurora, CO USA; 2 Colorado Center for Personalized Medicine, University of Colorado-Anschutz Medical Campus, Aurora, CO USA; 3 Department of Biomedical Informatics, University of Colorado-Anschutz Medical Campus, Aurora, CO, USA; 4 Section of Genetics and Metabolism, Department of Pediatrics, Children’s Hospital Colorado, Aurora, CO USA; 5 Department of Epidemiology & Population Health, Albert Einstein College of Medicine, Bronx, NY USA

**Keywords:** Polygenic risk scores, Polygenic scores, Hispanics, Latinos, Latin Americans, Personalized medicine, Genomic studies, Health disparities

## Abstract

Genome-wide association studies (GWAS) have allowed the identification of disease-associated variants, which can be leveraged to build polygenic scores (PGSs). Even though PGSs can be a valuable tool in personalized medicine, their predictive power is limited in populations of non-European ancestry, particularly in admixed populations. Recent efforts have focused on increasing racial and ethnic diversity in GWAS, thus, addressing some of the limitations of genetic risk prediction in these populations. Even with these efforts, few studies focus exclusively on Hispanics/Latinos. Additionally, Hispanic/Latino populations are often considered a single population despite varying admixture proportions between and within ethnic groups, diverse genetic heterogeneity, and demographic history. Combined with highly heterogeneous environmental and socioeconomic exposures, this diversity can reduce the transferability of genetic risk prediction models. Given the recent increase of genomic studies that include Hispanics/Latinos, we review the milestones and efforts that focus on genetic risk prediction, summarize the potential for improving PGS transferability, and highlight the challenges yet to be addressed. Additionally, we summarize social-ethical considerations and provide ideas to promote genetic risk prediction models that can be implemented equitably.

## Introduction

Hispanic/Latino is a common nomenclature used in epidemiological studies, likely because it is the nomenclature used by the US Census Bureau (United States Census Bureau 2022a). The terms “Hispanic” and “Latino,” as described by the US Office of Management and Budget and the US Census Bureau, are almost exclusively used in the USA, and they broadly refer to individuals from Latin America and/or Spanish-speaking countries (Lavange et al. 2010). Notably, while not primarily Spanish-speaking, Brazil is included in the “Latino” designation and Spain is often included as a “Hispanic” country. Additionally, this term is extended to the offspring of these individuals (Lavange et al. 2010) and individuals whose ancestors resided on modern US territory at a time that predated the establishment of the USA. Latin America has a complex demographic history, first occupied by Indigenous Americans then shaped by European colonization and the forced relocation of African individuals. The timing and magnitude of relocation have shaped the different admixture patterns and proportions of continental genetic ancestry throughout Latin America. Therefore, Hispanics/Latinos are known to be a genetically admixed population with unique demographic histories (Homburger et al. 2015).

Multiple studies have already documented the genetic admixture differences in Hispanics/Latinos (Bryc et al. 2015; Conomos et al. 2016). These differences are not only observed in the USA but also in Latin America as well (Belbin et al. 2018; Soares-Souza et al. 2018). For example, some individuals from South America have country-specific local patterns of genetic variation that are not observed outside certain sub-continental regions. Furthermore, Indigenous American and European ancestral components are significantly different throughout South American populations. Importantly, Indigenous American components are highly correlated with geography while European components are mainly from the Iberian Peninsula, even though some countries harbor components from Southern European regions (Homburger et al. 2015). On average, individuals from mainland backgrounds have higher Indigenous American ancestry proportions and lower African ancestry proportions than those from Caribbean countries. For instance, individuals from the Dominican Republic and Puerto Rico have a higher proportion of African genetic ancestry than those from Mexico or Central America (Bryc et al. 2015; Conomos et al. 2016). These different patterns of genetic ancestry impact variation in complex biomedical traits (Belbin et al. 2018)

Thousands of genetic variants associated with disease phenotypes and traits have been identified through genome-wide association studies (GWAS). As of September 2023, the GWAS catalog contained 6574 publications and 552,954 associations (Sollis et al. 2023). GWAS summary data is accessible through the catalog and can be leveraged to build polygenic scores (PGSs). Given the polygenicity of complex traits, PGSs aim to use individuals’ genomic data to make predictions of common complex diseases such as type 2 diabetes (T2D), asthma, and cardiovascular disease, among others. However, most genomic studies have historically focused on populations of European ancestry, limiting the utility of genetic risk prediction in populations that are genetically distant from most GWAS summary data (Kim et al. 2018). In the last decade, the US National Institutes of Health (NIH) has encouraged the inclusion of non-European participants in the genomic studies it funds. While this has led to an increase of studies that include populations of Asian ancestry, there has been minimal increase of Hispanics/Latinos and individuals of African ancestry. Importantly, these same populations that are underrepresented in genomic studies are also the ones traditionally underserved in the USA (Popejoy and Fullerton 2016). Therefore, the clinical implementation of PGSs constructed from European-ancestry GWAS summary data can potentially further deprive underserved communities from the benefits of personalized medicine.

Accuracy and portability of PGSs vary across global populations (Kim et al. 2018). To better understand the genetic architecture of complex biomedical traits in diverse populations, there have been increasing efforts to include ancestrally diverse populations in genomic studies. Among these efforts is the Population Architecture using Genomics and Epidemiology (PAGE) study. The goal of PAGE is to better understand the genetic susceptibility of disease in ancestrally diverse populations by collaborating with several US institutions, cohorts, and biobanks to conduct genetic analyses (Wojcik et al. 2019). However, one of the remaining limitations is the continued grouping of Hispanics/Latinos as a homogenous population, particularly in GWAS and polygenic risk prediction models. Additionally, Hispanic/Latino ethnic groups can have different disease rates and prevalence. For example, rates of asthma are approximately 3× higher in Puerto Ricans compared to Mexicans. The rate of asthma in Mexicans, in turn, is nearly 30% lower than the rate of asthma in non-Hispanic White individuals (US Department of Health and Human Services Office of Minority Health 2021). The vast genetic admixture diversity of Hispanics/Latinos (Conomos et al. 2016; Homburger et al. 2015), combined with different environmental factors, can lead to different health outcomes between ethnic groups (Belbin et al. 2018). Even though computational methods exist to account for population structure, challenges remain in the equitable implementation of PGSs in Hispanics/Latinos. Lastly, limited research has focused on the ethical, legal, and social implications of the use of genomic data in these populations and the subsequent clinical implementation of genetic risk scores. Throughout this review, we refer to these scores as polygenic scores or PGSs for short. Readers may be familiar with terms such as genetic risk scores, polygenic risk scores, or genetic risk prediction, which can all be viewed as a form of capturing the genomic contribution to a phenotype analogous to polygenic scoring.

## Using genomic data to understand disease susceptibility in Hispanics/Latinos: milestones

According to the 2020 US Census Bureau, Hispanics/Latinos are the largest growing minority group in the USA, making up approximately 18.7% of the total US population (Jones et al. 2021). Hispanics/Latinos experience higher rates of chronic diseases, notably diabetes and obesity, when compared to non-Hispanic Whites (U.S. Department of Health and Human Services Office of Minority Health 2021). Genomic biomedical research has focused on identifying the possible genetic factors driving disease, creating an opportunity for precision medicine intiatives to potentially address existing health disparities in Hispanics/Latinos. In this section, we review a few diseases burdening Hispanic/Latino populations to show how genomic data is being used to better understand disease etiology.

Type 2 diabetes (T2D) is highly prevalent in Hispanics/Latinos, with incidence rates almost twice as high compared to non-Hispanic whites (Aguayo-Mazzucato et al. 2019). With the exception of a few genomic studies focusing on Mexican-Americans and Mexicans (Below et al. 2011; Palmer et al. 2015; Parra et al. 2011), GWAS of T2D have mostly included individuals of European ancestry. In 2014, over 100 loci associated with T2D were identified through a trans-ancestry meta-analysis GWAS which included 40% of non-European individuals (DIAbetes Genetics Replication Meta-analysis Consortium, Asian Genetic Epidemiology Network Type 2 Diabetes Consortium, South Asian Type 2 Diabetes Consortium, Mexican American Type 2 Diabetes Consortium, Type 2 Diabetes Genetic Exploration by Nex-generation sequencing in multi-Ethnic Samples Consortium, et al. 2014). While this effort included almost half of the total individuals from non-European ancestry, only 2% were identified as Hispanic/Latino. More recently, a GWAS was conducted on individuals from six Hispanic/Latino ethnic groups (Central American, Cuban, Dominican, Mexican, Puerto Rican, South American) to search for novel disease-susceptibility loci and to better understand the genetic basis of T2D in Hispanic/Latino populations (Qi et al. 2017). The results led to the identification of an independent association signal shown to be African ancestry-specific in certain Hispanic/Latino ethnic groups. These findings support the importance of including Hispanics/Latinos in genomic studies to identify disease-associated variants that may appear in higher frequency in certain groups. The benefits of performing genetic analyses in diverse populations to identify novel loci and variants associated with complex traits have been well-documented (Sirugo et al. 2019; Wojcik et al. 2019; Young et al. 2018).

Asthma is a common complex disease that exhibits differential burden across Hispanics/Latinos. In the USA, Mexican-Americans have lower asthma prevalence than Puerto Ricans, who have the highest asthma burden across all populations. The variability of asthma prevalence and morbidity in different Hispanic/Latino ethnic groups has prompted research on these populations for the last two decades (Rosser et al. 2014). While the observed differences are likely multifactorial, some studies have focused on the genetic risk factors driving disease by identifying genetic variants that confer asthma susceptibility in highly burdened ethnic groups (Yan et al. 2017). Another effort towards advancing Hispanic/Latino representation in asthma genomic studies is a meta-analysis GWAS focusing on childhood-onset asthma from four independent and diverse cohorts (Yan et al. 2021). This study was prompted by the high burden of asthma complications in Latino youth resulting in increased school absences and healthcare costs. More specifically, the goal of the study was to identify genetic determinants of asthma exacerbations (e.g., hospital or clinical visits for acute care), which may be different than those of asthma itself. Yan et al. identified variants associated with asthma complications in Latino children and adolescents, thus, improving our genetic understanding of a common disease over-burdening certain Hispanic/Latino ethnic groups.

Another important study in Hispanics/Latinos was completed in 2014, specifically looking at cardiovascular risk factors in the Hispanic Community Health Study (HCHS), a Study of Latinos (SOL), often abbreviated as HCHS/SOL (Daviglus et al. 2014). This study summarizes the importance of clinical risk factors and how these may vary between Hispanic/Latino ethnic groups. The authors evaluate the lifestyle, socioeconomic, and sociocultural factors that can drive health outcome differences between ethnic groups. While these non-genetic risk factors are important to understand cardiovascular disease in Hispanics/Latinos, these are likely interacting with genetic risk factors and impacting other diseases as well. Similar studies have shown a higher risk of obesity and associated chronic diseases in Hispanics/Latinos, aiming to identify variants that may increase disease risk (Young et al. 2018). These research studies are a step forward in improving Hispanic/Latino health, yet many studies remain underpowered to properly identify population-specific variants. Therefore, the ethical recruitment and representation of Hispanics/Latinos from diverse ethnic backgrounds in genomic studies is imperative to better understand both genetic and non-genetic factors driving common complex diseases in these populations.

## Recruitment of Hispanics/Latinos in genomic studies is essential to address health disparities

Cohorts specifically designed to study Hispanic/Latino health are an important tool to address health disparities in these populations. HCHS/SOL is the largest community-based Hispanic/Latino cohort in the USA. This cohort involved the recruitment of diverse Hispanic/Latino ethnic groups (Cuba, Puerto Rico, Dominican Republic, Mexico, Central America, and South America) in four different communities in the USA (Lavange et al. 2010). Another notable Hispanic/Latino cohort is the Cameron County Hispanic Cohort (CCHC), which is a community-based cohort of approximately 5000 Mexican-Americans from a US-Mexico border community (The University of Texas Health Science Center at Houston 2019). Cohorts that specifically include Hispanic/Latino populations, such as HCHS/SOL and CCHC, provide an opportunity to potentially identify variants conferring disease risk in these populations. However, cohort heterogeneity, particularly when studied as a homogenous population, can create challenges that increase genomic inflation, confounding effects, and/or other effects such as relatedness that decrease statistical power (Conomos et al. 2016). Future research must continue to address the challenges of working with admixed populations such as Hispanics/Latinos and aim to develop statistical tools and methods that can account for heterogeneous variances between and within ethnic groups.

The increased number of Hispanics/Latinos in genomic and public health studies has provided unique opportunities to evaluate health disparities, however, few studies provide nationality (i.e., country of birth). Nationality is known to be an important healthcare factor with associated differences in health outcomes (Heintzman et al. 2023). The Multi-Ethnic Cohort Study (MEC), The Multiethnic Study of Atherosclerosis (MESA), and the Women’s Health Initiative (WHI) are some of the most diverse cohorts, which among others, are included in the PAGE consortium to better understand genetic susceptibility of disease in diverse populations. More recently, the *All of Us* research rogram is committing to enroll 1 million individuals with at least half from underrepresented or disadvantaged backgrounds (All Us Res Program Investigators 2019). As of September 2023, more than 710,000 participants have consented to join the All of Us research program, approximately 16% of them identifying as Hispanic/Latino. Notably, even with the increased number of Hispanics/Latinos in genomic studies, these only represent a small fraction of the total number of individuals enrolled, thus, still falling behind the power achieved in genomic studies of European ancestry such as is the case with the UK Biobank **(**Table 1**)**. These recruitment patterns are not only observed in some cohorts but in biobanks as well.

**Table 1:** Summary of cohorts and biobanks with genomic data that include Hispanics/Latinos

Name of cohort, study, or biobank	Year of initial recruitment	Recruitment location	Number of Hispanic/Latinos (approximate)	Nationality collected at initial recruitment	Percent of Latinos/Hispanics in cohort, study, or biobank	Reference(s)
Hispanic Community Health/Study of Latinos (HCHS/SOL)	2008	Miami, FL San Diego, CA Chicago, IL Bronx, NY	16,000	Yes	100%	(Lavange et al. 2010)
Cameron County Hispanic Cohort (CCHC) (The University of Texas Health Science Center at Houston, 2019)	2004	Brownsville, Texas Laredo, Texas	5,000	Yes	100%	(The University of Texas Health Science Center at Houston 2019)
Consortium for the Analysis of the Diversity and Evolution of Latin America (Candela)	2014	Brazil Chile Colombia Mexico Peru	7,300	Yes	100%	(Ruiz-Linares et al. 2014)
Mexico City Prospective Study	1998	Mexico City	150,000	Yes	100%	(Ziyatdinov et al. 2023)
Mexican Biobank	2000	Across 32 states in Mexico	6,000	Yes	100%	(Sohail et al. 2023)
Genetic Epidemiology Research on Adult Health and Aging (GERA)	2005	California	7,600	Yes	7.4%	(Banda et al. 2015)
Multi-Ethnic Cohort study (MEC)	1995	Majority California	18,400	No	24.2%	(The Multiethnic Cohort Study 2022)
Multi-Ethnic Study of Atherosclerosis (MESA)	2000	Baltimore City, MD Los Angeles, CA New York, NY St. Paul, MN	1,500	No	22%	(The Multiethnic Study of Atherosclerosis (MESA) n.d.)
Women’s Health Initiative (WHI)	1993	Across the USA	2,000	No	3%	(Women's Health Initiative 2021)
BioMe Biobank	2006	New York	18,400	Yes	33.8%	(The Charles Bronfman Institute for Personalized Medicine 2022)
Colorado Center for Personalized Medicine Biobank	2014	Colorado	18,100	No	9.2%	(Wiley et al. 2022)
UCLA ATLAS Precision Health Biobank	2016	California	3,900	No	14%	(Johnson et al. 2023)
Million Veteran Program	2011	Across the USA	78,800	No	8%	(Gaziano et al. 2016; U.S. Department of Veterans Affairs n.d.)
All of Us (All Us Res Program Investigators, 2019)	2016	Across the USA	79,000	Yes	16%	(All Us Res Program Investigators 2019)
UK Biobank	2006	United Kingdom	< 1,000	No	0.2%	(Zhou et al. 2022)

Columns capture year of initial recruitment, location of recruitment, approximate number of Hispanics/Latinos included, and the corresponding percentage that these populations represent in each respective study or biobank. Some of these studies are actively recruiting and numbers may change over time. List is not all-inclusive

Hispanics/Latinos make up approximately 24% of all participants in the Multi-Ethnic Cohort Study (MEC) and only 3% of the Women’s Health Initiative (WHI). In the context of Biobanks, we use the University of California Los Angeles ATLAS Community Health Initiative, abbreviated as UCLA ATLAS, as an example of Hispanic/Latino underrepresentation. UCLA ATLAS recruits participants from across the UCLA Health system in the greater Los Angeles area (Johnson et al. 2023). Even though LA County has one of the largest Hispanic/Latino populations in the country, where 49% of individuals identify as Hispanic/Latino (United States Census Bureau 2022b), only 14% of participants in the UCLA ATLAS Precision Health Biobank self-identified as Hispanic/Latino (Johnson et al. 2023). The continued underrepresentation of Hispanics/Latinos, combined with a lack of genomic research addressing the heterogeneity of Hispanic/Latino groups who have different cultures and environmental factors, remains a challenge in extending the benefits of personalized medicine to these populations. However, it is important to note ongoing active consortium-based and national initiatives occurring across Latin America, including novel, large studies coming online such as the Mexico City Prospective Study (Ziyatdinov et al. 2023), the Mexican Biobank (Sohail et al. 2023), and the trait-focused consortia Candela (Ruiz-Linares et al. 2014). These efforts both build capacity and collaboration for international researcher teams and improve our understanding of genetics across Latin America, thus, improving our knowledge of genetic architecture patterns specific to Hispanics/Latinos.

## Current state of polygenic scores (PGSs) in Hispanics/Latinos

Historically, risk prediction models have focused on factors such as age, sex, family history of disease, and lifestyle. The promising advances of GWAS, particularly as studies become more diverse, have led to increased interest in predicting disease risk, onset, and outcomes from genomic data. Polygenic scores (PGSs) are traditionally calculated by aggregating the effects of disease-associated Single Nucleotide Polymorphisms (SNPs) from GWAS results (Choi et al. 2020). The GWAS summary statistics used to construct PGSs are referred to as the discovery sample; these developed PGSs are then evaluated in an independent sample (Wray et al. 2013). Because most GWASs are performed on individuals from European descent (Popejoy and Fullerton 2016), the PGSs developed from GWAS summary data (i.e., discovery samples) have a small number of Hispanics/Latinos. This limited and disproportionate sample size hinders the portability of PGSs in these populations (Kim et al. 2018). Therefore, PGSs that are developed using discovery samples from European individuals and applied to other populations, such as Hispanics/Latinos, have limited prediction accuracy (Bitarello and Mathieson 2020; Martin et al. 2017; Martin et al. 2019). The limitations of using disease SNP associations discovered in one population to predict disease in another population have been well-documented (Scutari et al. 2016; Wang et al. 2020; Wray et al. 2013).

Polygenic risk score prediction accuracy decreases in non-Europeans, particularly those individuals who are more genetically distant from Europeans. These limitations are not only a result of differences in risk allele frequencies and specific loci relevant to different populations (Kim et al. 2018) but also differences in cross-population correlations of causal SNP effects and heritability (Wang et al. 2020). Importantly, we note that the SNPs identified through GWAS are not usually the causal variants of a particular phenotype or disease. Instead, these are identified due to their association with a phenotype of interest and may be highly correlated with one or more causal variants. The correlation between the associated variant and the causal variant(s) depends on linkage disequilibrium, or genetic regions that are inherited together and persist through generations, resulting in SNPs that are highly correlated to the development of a particular disease. Different populations have different linkage disequilibrium patterns, a biological characteristic known to limit the prediction accuracy of PGSs in underrepresented populations (Wang et al. 2020; Wray et al. 2013). Additionally, the effect sizes calculated from GWAS summary data are estimated based on the discovery sample, biasing allele effect sizes that more accurately represent European individuals due to their over-representation in GWAS (Popejoy and Fullerton 2016). For these reasons, the inclusion of Hispanics/Latinos in GWAS to identify population-specific loci and effect sizes, and their subsequent incorporation in PGSs can increase risk prediction accuracy in these populations.

The complex demographic history of Hispanics/Latinos has shaped the landscape of clinical variants both in Latin America as well as Hispanics/Latinos living in the USA. This history has created conditions that have led to variants that segregate in specific Hispanic/Latino groups. Some examples include highly-penetrant founder mutations in *BRCA1* and *BRCA2* in germline cases of Mexico, and Caribbean-specific founder variants in *PSEN1* genes that confer a risk of early-onset Alzheimer’s disease (Belbin et al. 2018). These population-specific variants, combined with genetic heterogeneity between and within Hispanic/Latino groups, highlight the importance of fine-scale population structure in the context of medical genetics in Hispanics/Latinos. In fact, fine-scale population structure affects polygenic risk prediction even in European populations that do not have the same complex demographic history and admixture of Hispanics/Latinos. For example, a recent study evaluated 245 PGSs in nine ancestry groups at a sub-continental level in the UK biobank. Their findings provide further evidence of the PGS portability problem even when working with European subpopulations (Prive et al. 2022). The authors further emphasize the relationship between genetic distance and predictive performance, even when PGSs are derived and applied in the same cohort. Therefore, it is important that PGSs are derived, trained, and evaluated in Hispanics/Latinos. Even in a large genomic database such as the UK Biobank, there is a low number of individuals that are identified as Hispanic/Latino, limiting the use of this database for these populations.

## Polygenic score (PGS) challenges and future directions

**Fig. 1:**
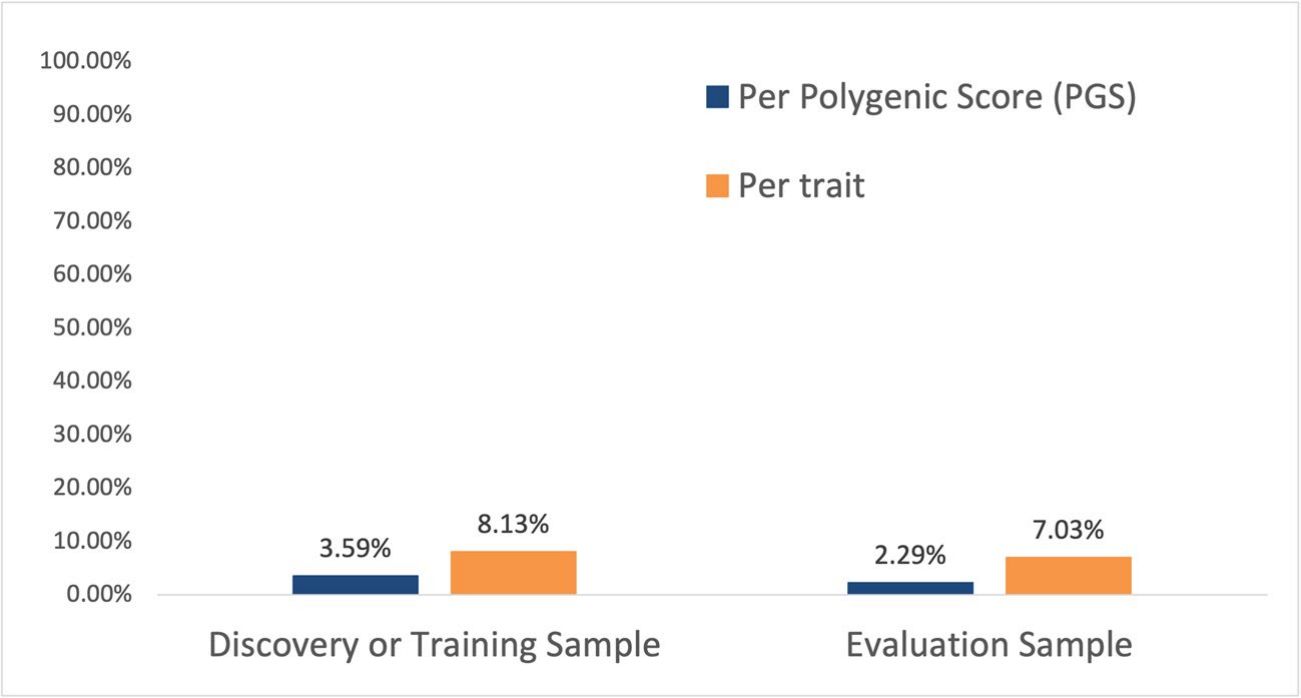
Percent of Hispanics/Latinos included in the Polygenic Score (PGS) Catalog. Estimation of Hispanics/Latinos included in discovery and/or training and evaluation samples in the Polygenic Score Catalog (PGS). Percentages were calculated by totaling the number of either polygenic scores (PGSs) or traits that specifically included the term Hispanics or Latinos in either discovery or training samples, and evaluation samples

One of the leading challenges in the implementation of polygenic scores (PGSs) in Hispanics/Latinos is the lack of representation of relevant populations in PGS development, training, and evaluation. As of September 2023, the PGS catalog had 3787 polygenic scores across 640 traits in 492 publications (Lambert et al. 2021). Of these 3787 published polygenic risk scores, only 136 (3.59%) had a discovery or training sample that included Hispanics/Latinos; the number is lower for those that included Hispanics/Latinos in the evaluation sample (87 total, 2.29%). When evaluating traits, only 52 of 640 traits included Hispanics/Latinos in the discovery or training sample (8.13%). This number is less when looking at the percentage of traits that included Hispanics/Latinos in the evaluation sample (45 out of 640 total traits, totaling 7.03%) (Fig. 1). Note that these estimates are derived from the PGS catalog metadata, which we used to determine the number of polygenic risk scores and traits that had Hispanics/Latinos specifically listed in PGS development, training, and evaluation samples. Importantly, the inclusion of Hispanics/Latinos only represents a fraction of the total number of individuals included in either a discovery, training, or evaluation sample. For example, the median percent of Latinos/Hispanics included in discovery samples is only 2.2% of the total number of individuals included. This indicates that the number of Hispanics/Latinos included in either polygenic risk score development, training, or evaluation sample is a small fraction of the total number of individuals in the development of PGSs. This poses a remarkable challenge in the clinical implementation of PGSs in these populations because increasing genetic distance between training and target populations decreases prediction accuracy (Scutari et al. 2016). However, these findings are not surprising given the underrepresentation of Hispanics/Latinos in GWAS which was less than 1% in 2016 (Popejoy and Fullerton 2016) and 1.13% in 2019 (Sirugo et al. 2019).

One of the most notable examples of using Hispanics/Latinos to increase the predictive power of PGSs was completed in 2020, aiming to improve genetic risk prediction of breast cancer in Latinas (Shieh et al. 2020). Even though the discovery of breast cancer-associated SNPs has been predominantly in European populations, some variants have been discovered in Latinas (Fejerman et al. 2014; Hoffman et al. 2019). Shieh et al. examined the performance of a PGS consisting of SNPs identified in European populations when adding those identified in Latinas. The authors concluded that the PGS with both European- and Latina-specific variants increased the predictive value in Latinas. Even though the authors show that this refined PGS has similar prediction accuracy in Europeans and across various Hispanic/Latino groups, this study highlights the importance of incorporating population-specific variants and evaluating risk prediction models to develop PGSs that maximize performance, particularly in underrepresented populations. However, one limitation is that this study only improved the predictive power of the trait studied (breast cancer), so the authors caution the over-generalization of this PGS to Latinas with different genetic ancestry proportions, as their findings are limited to the Latina ethnic group studied and those with similar distributions of genetic ancestry. Because demographic history impacts genetic risk prediction, to maximize PGS prediction accuracy, it is optimal to compute polygenic risk scores using discovery samples with similar demography history as the target population (Martin et al. 2017). This limitation is particularly pronounced in Hispanic/Latino populations who have varying genetic ancestry proportions which create further challenges in prediction accuracy.

The challenges in genetic risk prediction resulting from a lack of representation in Hispanics/Latinos are exacerbated by the lack of consistency in electronic health record (EHR) data and environmental factors that are difficult to quantify and measure, particularly in health system-associated biobanks. Studies have illustrated racial and ethnic differences in accessibility of patient portals that are linked to EHR data (Chang et al. 2018), especially impacting individuals of non-European ancestry and those with Spanish language preference (Garrido et al. 2015). The challenges that participants encounter when accessing patient portals are likely to affect phenotype harmonization, which is a key step in genomic studies and polygenic risk scoring. Another major concern is being able to fully understand genetic susceptibility in a society where access to healthcare significantly contributes to health disparities. A recent review of Hispanics/Latinos with type 2 diabetes found that cost, time, and lack of social support are among the barriers that prevent these populations from seeking and receiving the care they need (Titus and Kataoka-Yahiro 2021). Research conclusions must be carefully phrased to avoid claims that a certain population is more genetically predisposed than others, particularly because health risks can be compounded and such claims could have social and legal implications, especially for disadvantaged communities. To minimize bias in genomic research, scientists must be aware of the complex and layered risk factors that increase disease burden in Hispanic/Latino populations.

Lastly, a major limitation of PGSs is the inability to properly account for environmental factors that could be interacting with and driving risk beyond that of genomic data. For example, socioeconomic factors that affect Hispanics/Latinos may operate differently for undocumented immigrants compared to their documented counterparts, who may have higher accessibility to health care and health insurance (Cabral and Cuevas 2020). These challenges also affect disease diagnosis, which is associated with higher education completion and English literacy (Fisher-Hoch et al. 2012). To inform the clinical use of PGS-based risk stratification, it will also be necessary to examine the value of genetic risk prediction models in comparison to (or in addition to) established risk prediction equations based on clinical factors. This presents a further challenge, as even in fields such as preventive cardiology where clinical risk prediction approaches are well developed, the clinical (non-genetic) risk prediction equations have not been appropriately tailored to Hispanic/Latino populations due to lack of representation (Flores Rosario et al. 2021). The variability within Hispanic/Latino populations, both in genetic heterogeneity between and within ethnic groups, combined with a lack of representation in PGS samples and layered environmental factors, highlights the need for further research in the equitable clinical implementation of PGSs.

## Clinical utility of polygenic scores (PGSs) and ethical considerations

Our enhanced understanding of the genetic etiology of complex diseases has propelled a wave of research that aims to integrate this information into risk prediction models that can be used in clinical settings. Overall, one of the goals of these precision medicine efforts is to identify individuals that may benefit from prompt preventative or treatment measures. However, researchers have already cautioned the scientific community of potentially exacerbating health disparities if PGSs are implemented without addressing the aforementioned challenges (Martin et al. 2019). These concerns have led to the increased recruitment of historically underrepresented populations in genomics; however, it is important that these recruitment efforts follow ethical guidelines already set forth by multiple organizations such as the Global Alliance for Genomics and Health (Knoppers 2014) and the US National Commission for the Protection of Human Subjects of Biomedical and Behavior Research (Sims 2010), just to name a few. Among these guidelines are ensuring that this research is benefiting the populations studied, as well as promoting health, well-being, and fair distribution of benefits (Mudd-Martin et al. 2021).

Notably, some PGSs are already being implemented in clinical settings, yet, these are likely to provide greater benefit and predictive accuracy to European populations. Some existing commercial and clinical PGS products were based on data from GWAS catalogs that were only initially available to and validated in individuals of European descent, which inherently hinders their application to underrepresented populations. For example, Myriad’s riskScore which assesses breast cancer risk was initially validated in 2017 only for women of European ancestry (Preston 2017). Therefore, for approximately 4 years, this test was only available to women of European descent, excluding entire populations that could have benefited from this type of risk assessment, particularly those from underserved communities, which includes Hispanics/Latinos. Myriad subsequently received criticism for this, and a revised riskScore applicable to women of all ancestries was released 4 years later in 2021 (Ray 2021). Myriad’s riskScore clearly highlights how the underrepresentation in genomic studies has the potential to exacerbate health disparities through the lack of access to genomic advances and technologies. This example also illustrates how the pattern of developing and validating assays first in European cohorts without careful consideration of their social implications can be harmful, and even when followed by inclusion of non-European cohorts, which can happen several years later, is ethically problematic. While the recruitment of Hispanics/Latinos in genomic studies and biobanks is a sensible next direction, there are still challenges to be addressed. For example, participant basic survey questions that aim to capture race and ethnicity as a single category can be confusing to answer for many individuals that identify as Hispanic/Latino (Allen Jr. et al. 2011). The specific questions asked can create heterogeneity in the use of race and ethnicity terms across genomic studies. Likewise, further guidance is needed to ethically capture important environmental factors such as acculturation and a proxy for documentation status, as well as language accessibility for both recruitment and dissemination of results.

It is also important to evaluate the disparities within the public health system, as these may pose potential barriers to the equitable implementation of PGSs in Hispanics/Latinos—we use the COVID-19 pandemic as a relevant case in point. The COVID-19 pandemic revealed major shortcomings embedded in the public health system, resulting in underrepresented communities bearing the highest risk of COVID-19 morbidity and mortality (Dalva-Baird et al. 2021). Even though access to health care played a major role in this outcome, other studies highlighted additional factors that contribute to the hesitancy of certain groups to engage in preventative methods, such as COVID-19 vaccines. A recent study explored these barriers in the context of COVID-19 vaccination (Butler et al. 2022); however, these are likely challenges that will burden Hispanic/Latino populations in the clinical implementation of PGSs as well. Among these findings are unclear information, language barriers, inadequate exposure to trusted sources of information, and uncertainty in eligibility to receive personalized medicine benefits (i.e., health insurance). Therefore, the utility of PGSs as a preventative tool can be hindered by accessibility barriers that are specific to Hispanics/Latinos and limit their benefit in clinical settings. Future research should focus on developing a framework that actively involves the community, evaluates participant perspectives, identifies challenges, and addresses them appropriately to maximize the benefits of personalized medicine efforts in clinical settings.

Recent research has captured the perspectives of diverse Spanish- and English-speaking patients on the clinical use of PGSs (Suckiel et al. 2022). The study recruited 30 biobank participants ages 35–50 years that were either self-reported as African/African American or Hispanic/Latino. These participants were part of semi-structured interviews in Spanish and English that explored their attitudes towards PGSs. Despite being told about the limited predicted power of PGSs for non-European populations, participants indicated that high-risk scores would prompt them to follow-up with their physicians and implement healthy behavior changes. They also mentioned a few barriers for the adoption of PGS-related recommendations, among these are insurance status, language, and inadequate understanding of PGSs. Larger studies that capture Hispanic/Latino attitudes towards PGSs are needed to address these challenges and ensure their equitable implementation. As highlighted earlier, in any genomic study, it is important to caution patients, physicians, and scientists from assuming that one population group is more genetically predisposed to disease versus another, as genetic risk prediction is only part of the multiple factors conferring disease risk, particularly in underrepresented populations (Belbin et al. 2021). Risk assessment should instead be performed on an individualized basis, accounting for the appropriate PGS (if/when clinically available), and relevant clinical and environmental risk factors.

## Summary

The availability of genomic data has led to a rapid increase in Genome-Wide Association Studies (GWAS). Trait-associated SNPs, leveraged from the GWAS catalog, can be aggregated and used to make predictions of complex traits from individual genomic data. While recent efforts have increased the diversity of genomic studies, particularly in diseases that burden Hispanics/Latinos, lack of representation of Hispanics/Latinos in the development, training, and evaluation of polygenic scores (PGSs) remains a challenge. This lack of representation, combined with highly heterogeneous genetic, environmental, and socioeconomic exposures, affects the utility and accuracy of polygenic risk prediction in these populations. The limitations highlighted in this review can exacerbate existing health disparities and bring attention to the ethical, legal, and social implications of the clinical implementation of PGSs in Hispanics/Latinos.
